# Vestibular schwannoma presenting as vestibular neuritis-like attack during the COVID-19 pandemic: A case report

**DOI:** 10.1097/MD.0000000000045671

**Published:** 2025-10-31

**Authors:** Xiaoye Chen, Shan Chen, Ping Lei, Yingzhao Liu, Xingqian Shen, Kaijun Xia, Qin Liu, Ziying Xu, Hongjun Xiao, Bo Liu

**Affiliations:** a Department of Otorhinolaryngology-Head and Neck Surgery, ENT Institute, Union Hospital, Tongji Medical College, Huazhong University of Science and Technology, Wuhan, China; b Hubei Province Clinical Research Center for Deafness and Vertigo, Wuhan, China; c Department of Radiology, Union Hospital, Tongji Medical College, Huazhong University of Science and Technology, Wuhan, China.

**Keywords:** acute vestibular syndrome, vertigo, vestibular function, vestibular neuritis, vestibular schwannoma

## Abstract

**Rationale:**

Vestibular schwannoma (VS) is a benign tumor commonly presenting with progressive hearing loss, tinnitus, and disequilibrium. However, in rare cases, it can mimic acute vestibular syndromes such as vestibular neuritis. Early recognition of such atypical presentations is essential for accurate diagnosis and timely management.

**Patient concerns:**

A 54-year-old woman experienced a sudden onset of sustained vertigo lasting 2 days without accompanying hearing loss or tinnitus. She had no prior history of vertigo.

**Diagnoses:**

Neurotological assessments showed direction-fixed, horizontal-torsional nystagmus. Vestibular function tests revealed right-sided canal paresis on caloric testing and reduced vestibulo-ocular reflex gains across all right semicircular canals. Audiometry showed symmetrical, mild high-frequency hearing loss. Brain magnetic resonance imaging identified a 0.4 cm intracanalicular lesion consistent with VS.

**Interventions:**

The patient received oral corticosteroids, betahistine, ginkgo biloba extract, and vestibular rehabilitation therapy.

**Outcomes:**

Vertigo symptoms improved within 2 weeks and resolved completely by 5 months. No recurrence of vertigo was observed during a 2-year follow-up. Hearing remained stable, and no surgical intervention was required due to the small tumor size.

**Lessons:**

VS can present with acute vestibular syndrome resembling vestibular neuritis, even in the absence of auditory symptoms. Comprehensive neurotological evaluation and high-resolution magnetic resonance imaging are crucial for differential diagnosis and management.

## 1. Introduction

Vestibular schwannoma (VS) is a benign tumor that develops from the Schwann cells surrounding the vestibular branch of the eighth nerve.^[1]^ As the tumor enlarges, it can lead to compression and damage to the nerve and adjacent structures, causing symptoms such as progressive hearing loss, tinnitus, disequilibrium, vertigo, and facial numbness.^[2]^ Vertigo occurs in approximately 10% to 19% VS cases, while the frequency, severity and progression of vertigo can vary widely.^[3]^ For instance, Sahyouni et al^[4]^ have reported vestibular migraine and benign paroxysmal positional vertigo are underlying factors for vertigo in VS. Also, 4% to 5% VS patients may present symptoms that mimic Meniere’s disease (MD) in earlier studies.^[5]^ However, the attack of acute vestibular syndrome (AVS) for patients with VS was rarely reported. Here, we report a case of VS patient mimicking vestibular neuritis (VN) by the clinical presentation of AVS during the period of pandemic.

## 2. Case presentation

**Figure 1.**
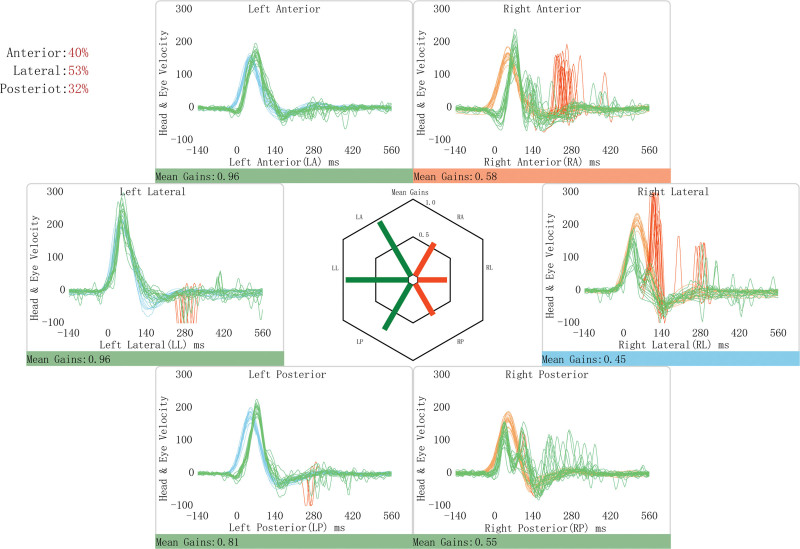
The vHIT results of the 54-year-old female with VS. The cupulogram (hexagon in the center of the figure) confirms a decrease in the gain for 3 right semicircular canals. Head and eye velocity curves confirm the decrease in the VOR gain for the right anterior and lateral canals, and demonstrate the presence of catch-up saccades (red spikes). Normal gain in all 3 left SCCs indicating acute right unilateral vestibular loss. SCCs = semicircular canals, vHIT = video head impulse test, VOR = vestibulo-ocular reflex, VS = vestibular schwannoma.

**Figure 2.**
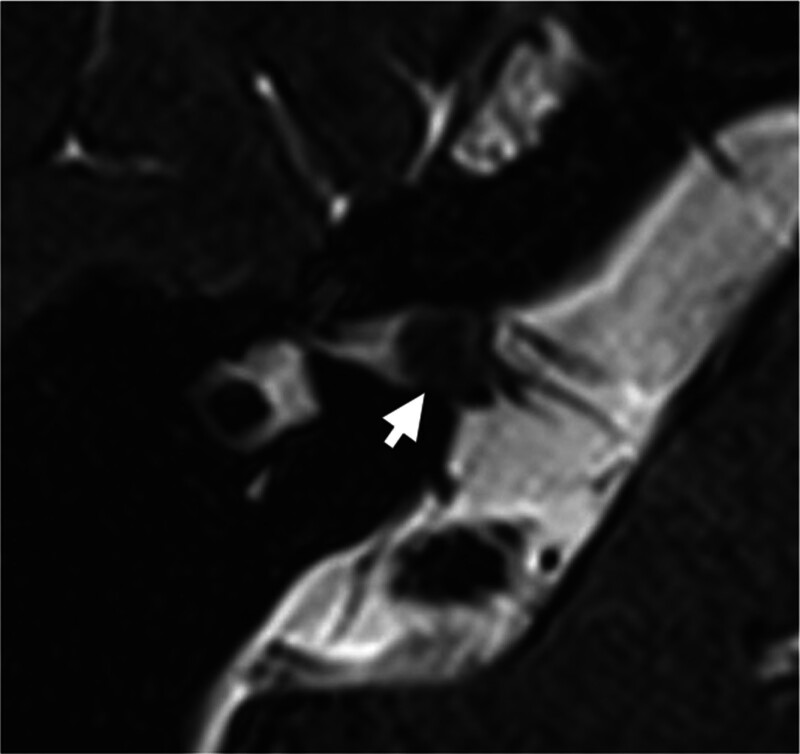
Axial 3-dimensional sampling perfection with application optimized contrasts using different flip-angle evolutions (3D-SPACE) image of the patient showing small intracanalicular vestibular schwannoma located in the right internal auditory canal. 3D-SPACE = 3-dimensional sampling perfection with application optimized contrasts using different flip-angle evolutions.

A 54-year-old female patient attached to our department in December 2022 for acute onset of rotatory vertigo for 2 days, without hearing loss or tinnitus. The patient never experienced vertigo before and received emergency treatment for dizziness at local hospital. After we ruled out the possibility of central vestibular lesions based on the lack of brain stem or cerebellar symptoms along with the negative result in cranial magnetic resonance imaging (MRI), the patient underwent a Head-Impulse-Nystagmus-Test-of-Skew examination in our department. The preliminary diagnosis was right VN. The patient had left-beating, horizontal-torsional, direction-fixed nystagmus obeying Alexander’s law. The pure tone average (PTA) revealed symmetry mild high frequency sensorineural hearing loss at 125 Hz to 8 kHz on both sides. The results of caloric test indicated 100% right canal paresis. Bilateral and symmetrical air-conducted cervical vestibular evoked myogenic potentials (cVEMP) and ocular vestibular evoked myogenic potentials were evoked by air-conducted stimulation. The video head impulse test (vHIT) was conducted using the ICS Impulse system (GN Otometrics, Denmark) by an experienced technician, and the results showed reduced gain for all 3 right semicircular canals (a horizontal vHIT gain < 0.8 or a vertical vHIT gain < 0.7) with scattered covert and overt saccades (Fig. 1). 3-dimensional sampling perfection with application optimized contrasts using different flip-angle evolutions (3D-SPACE) image revealed a 0.4 cm tumor in the right internal auditory canal, considering to the diagnose with VS (Fig. 2).

The patient received oral corticosteroids, betahistine, and ginkgo biloba extract, along with vestibular rehabilitation therapy. As a follow-up for 2 weeks, the patient reported improved vertigo symptoms. However, the dizziness remained during rapid head movements and lying down, and nystagmus was also evoked by positional testing. Although continued vestibular rehabilitation therapy was recommended, the patient did not attend subsequent visits. Follow-ups conducted via calls at 3 months, 6 months, 1 and 2 years revealed no recurrence of severe vertigo episodes. After treatment for 5 months, the symptoms of imbalance and disequilibrium during activity gradually improved to normal state. Also, the patient did not report further hearing deterioration. Due to the small size of the tumor, neither surgical intervention nor stereotatic radiosurgery was performed. The patient was advised to undergo follow-up MRI and audiovestibular function reassessments and remain under periodic monitoring.

The study was approved by the Ethical Committee of Union Hospital, Tongji Medical College, Huazhong University of Science and Technology (Institutional Review Board approval No. 0153, 2025.2.22). Written informed consent was obtained from the patients to publish this paper. This case report adheres to the CARE reporting guidelines for presentation.

## 3. Discussion

VS typically manifests as episodic vestibular syndrome and presents hearing loss, tinnitus, and vertigo as a chronic and progressive disorder,^[3,4]^ but patients are rarely present as AVS. In the present case, the patient exhibited negative central nervous system signs, and acute central lesions were ruled out by MRI. Additionally, sudden sensorineural hearing loss was excluded by symmetry PTA. Based on the manifestation of AVS, nystagmus, vestibular function tests and cranial MRI, the case also satisfied the diagnostic criteria for VN/acute unilateral vestibulopathy in accordance with the guidelines.^[6]^

Similar rare presentations have been documented in only 2 reports. Sunara et al^[7]^ reported a case previously diagnosed as VS developed symptoms of AVS, and accompanied by hearing loss and tinnitus. They suggested that VN may overlap the vestibular nerve, which already damaged by VS. In addition, Park et al^[8]^ demonstrated a VS patient who had AVS but no tinnitus or hearing loss, which was similar to our study. This patient exhibited mild canal paresis on the left side in caloric test, significantly lower gains on the ipsilateral horizontal and anterior canals on vHIT, and absent on cVEMP, indicating the impaired vestibular function on the left. The patient in our investigation also exhibited significantly reduced mean vestibulo-ocular reflex gains with saccades in all 3 semicircular canals. Previous studies reported abnormal cVEMP and ocular vestibular evoked myogenic potentials rates ranging from 38.5% to 64.4%, and 15.4% to 68.9% respectively in VS.^[9,10]^ The tiny tumor size (4 mm) may account for the normal VEMP results in our study. Furthermore, Taylor et al^[11]^ found significant correlations between vestibular test results and tumor size, demonstrating that patients with schwannomas smaller than 14 mm had no vestibular abnormalities, indicating that smaller tumors are associated with fewer abnormal vestibular findings.

There are a number of possible causes for vertigo in VS. Studies have reported that 16.7% to 30.8% VS patients exhibited endolymphatic hydrops in MRI on the affected side.^[12]^ Recently, Wang et al^[13]^ have found that the area and volume of vestibular endolymph on the affected side were noticeably larger than the contralateral side. Thus, vertigo and MD-like symptoms in VS could be attributable to endolymphatic hydrops. In addition, episodic vestibular syndrome due to comorbidities like benign paroxysmal positional vertigo and vestibular migraine in VS have been reported by Sahyouni et al.^[4]^ Furthermore, the compression and metabolic abnormalities caused by the schwannoma may result in injury to the vestibular end organs. Potential mechanisms of episodic vertigo have been described including the blockage of the neuroaxonal transport of proteins by compression of the nerve, the impairment of labyrinthine blood flow, the cell-mediated immune response to the inner ear caused by the tumor antigens, and the release of toxins or potassium ions by the tumor.^[14]^

VS can present acute sudden sensorineural hearing loss in 7% to 26% cases,^[15]^ and earlier studies showed that 0.8% to 47.5% of the patients with sudden sensorineural hearing loss diagnosed as VS.^[16]^ However, the symmetry PTA allows differentiation from sudden deafness associated with vertigo in this patient. The possible explanation for the onset of AVS in our patient is the affected ear has both VS and VN, which could have been induced by a viral infection, particularly considering the period of the coronavirus disease 2019 (COVID-19) pandemic. Interestingly, the onset of vertigo in December 2022 was just the time overlap of the COVID-19 reopening policy. A study from Wuhan has demonstrated that 16.8% confirmed patients of COVID-19 present vestibular symptoms,^[17]^ and VN has been reported as a clinical presentation of COVID-19 in several reports.^[18]^ Through anosmia, ageusia, SSNHL and facial palsy, COVID-19 has proven its neurotropic and neuroinvasive features. Our report notes that unilateral VS with COVID-19 infection may facilitate the neuro-invasive propensity. Hypotheses of the lesion may relate to the acute ischemia and compression of the vestibular nerve by VS, and the infection could also enhance the thrombus formation in the blood vessels leading to ischemia damage and the onset of VN. Moreover, the inflammatory process after infection associates with the ischemia and demyelination could also generate VN.^[18]^ Other potential causes include the syndromes present as the initial vertigo episode of VS that resembling MD, the acute ischemia and the compression of the vestibular nerve. Overall, neuroimaging is crucial to exclude central lesions and space-occupying pathologies in patients presenting with AVS, in accordance with the diagnostic criteria for VN. Our patient’s 2 years follow-up without vertigo episodes further highlights the significance of a comprehensive imaging evaluation for AVS.

## 4. Conclusion

This study presents a case of VS manifesting as AVS without hearing loss during period of the COVID-19 pandemic, confirmed through neurotological evaluation and MRI, while its etiology warranting further investigation. In conclusion, neurotological examinations are essential for the diagnosis and management of patients with AVS.

## Acknowledgments

The authors have reviewed and edited the output and take full responsibility for the content of this publication.

## Author contributions

**Conceptualization:** Bo Liu.

**Formal analysis:** Yingzhao Liu, Xingqian Shen, Kaijun Xia, Qin Liu, Ziying Xu.

**Investigation:** Xiaoye Chen, Shan Chen, Ping Lei, Bo Liu.

**Methodology:** Shan Chen, Ping Lei, Bo Liu.

**Resources:** Ping Lei, Bo Liu.

**Supervision:** Hongjun Xiao, Bo Liu.

**Visualization:** Xiaoye Chen.

**Writing – original draft:** Xiaoye Chen, Shan Chen, Yingzhao Liu, Xingqian Shen, Kaijun Xia, Qin Liu, Ziying Xu.

**Writing – review & editing:** Xiaoye Chen, Hongjun Xiao, Bo Liu.
